# Serum biomarker levels in smokers and non-smokers following periodontal therapy. A prospective cohort study

**DOI:** 10.1186/s12903-024-04196-8

**Published:** 2024-04-16

**Authors:** Lorenz V. Knie, Knut N. Leknes, Ying Xue, Stein Atle Lie, Dagmar F. Bunæs

**Affiliations:** 1 Department of Clinical Dentistry, Faculty of Medicine, University of Bergen, Årstadsveien 19, Bergen, N-5009 Norway; 2 Oral Health Centre of Expertise Rogaland, Stavanger, Norway; 3 Faculty of Health Sciences, Department of Clinical Dentistry, UiT The Arctic University of Norway, Tromsø, Norway

**Keywords:** Periodontitis, Smoking, Periodontal treatment, Systemic inflammation

## Abstract

**Background:**

To compare presence and levels of serum cytokines in smokers and non-smokers with periodontitis following periodontal therapy.

**Methods:**

Thirty heavy smokers and 30 non-smokers with stage III or IV periodontitis were included in this prospective cohort study. Clinical data and blood serum were collected at baseline (T0), after step I-III (T1), and after 12 months step IV periodontal therapy (T2). Cytokine IL-1β, IL-6, IL-8, TNF-α, IL-10, and IP-10 levels were measured using multiplex kit Bio-Plex Human Pro™ Assay. Linear regression models with cluster robust variance estimates to adjust for repeated observations were used to test intra- and intergroup levels for each marker, IL-6 and IL-8 defined as primary outcomes.

**Results:**

Clinical outcomes improved in both groups following therapy (*p* < 0.05). IL-6 levels increased with 75.0% from T0-T2 among smokers (*p* = 0.004). No significant intra- or intergroup differences were observed for IL-8. Higher levels of TNF-α (44.1%) and IL-10 (50.6%) were detected in smokers compared with non-smokers at T1 (*p* = 0.007 and *p* = 0.037, respectively). From T1-T2, differences in mean change over time for levels of TNF-α and IL-10 were observed in smokers compared with non-smokers (*p* = 0.005 and *p* = 0.008, respectively).

**Conclusion:**

Upregulated levels of serum cytokines in smokers indicate a systemic effect of smoking following periodontal therapy. Differences in cytokine levels between smokers and non-smokers demonstrate a smoking induced modulation of specific systemic immunological responses in patients with severe periodontitis.

**Supplementary Information:**

The online version contains supplementary material available at 10.1186/s12903-024-04196-8.

## Background

Periodontitis is associated with low-grade systemic inflammation [1, 2]. Major mechanisms in the pathogenesis appear to be an altered host response to subgingival microbiota [3]. Periodontal pathogens induce release of inflammatory markers orchestrating innate and adaptive immune responses. The balance and interactions among these molecules determine whether the inflammatory response remains stable or provokes disease [4, 5]. In patients with periodontitis, inflammatory cytokines in serum and gingival tissues are upregulated and may increase the risk of developing cardio-metabolic diseases due to common pathogenetic mechanisms [6–8].

A network of cytokines modulates the inflammatory processes and homeostasis acting in the first line of defense against periodontal pathogens [9]. Bacterial pathogens trigger the host immune response which causes release of inflammatory mediators and cytokines that play an important role in the pathobiology of periodontitis [10]. Increased levels of proinflammatory cytokines including Interleukin IL-1 β (IL-1 β), Interleukin 6 (IL-6), Interleukin 8 (IL-8), Tumor Necrosis Factor-α (TNF-α), and regulatory cytokines including Interleukin 10 (IL-10), and Interferon gamma-induced protein (IP)-10 (or CXCL-10) have been shown in patients with periodontitis [11]. IL-6 has multiple members and pleiotropic functions in the immune response, hematopoiesis, bone metabolism, and cancer. Further, IL-6 is involved in the pathogenesis of periodontitis [9]. IL-8 is a potent chemoattractant and activator and is produced in both infectious and inflammatory diseases. In the inflamed periodontium, IL-8 is produced by activated polymorphonuclear leukocytes, epithelial cells, and gingival fibroblasts [12].

Cigarette smoking has a downregulatory effect on humoral and cell mediated immunity through a negative impact on the expression on several genes [13]. The immune system in smokers displays signs of dysfunction including increased tendency to autoantibody production, reduced polymorphonuclear neutrophil chemotaxis, and phagocytic capabilities [14]. The conservation of pro- and anti-inflammatory cytokines in serum in non-smoking and smoking periodontitis patients indicate an overall proinflammatory cytokine imbalance [15].

Periodontal therapy may reduce systemic inflammation, especially in subjects with systemic comorbidities including diabetes and metabolic syndrome [16]. During periodontal therapy, systemic inflammation is affected by an acute phase inflammatory response which is followed by a long-term remission [17]. Non-surgical periodontal therapy decreases periodontal inflammation locally, but overall changes in systemic inflammation may also be observed including reduction in C-reactive protein (CRP), IL-6, and IL-8 [18]. A randomized controlled trial evaluating the effect of periodontal therapy on cardiovascular biomarkers observed overall changes in systemic inflammation specifically for IL-6 and IL-8. Levels of IL-6 and IL-8 were significantly reduced in the test group receiving step I and II periodontal therapy compared with control receiving a session of dental plaque removal [18]. Similarly, a prospective cohort study investigating the effect of heavy smoking on inflammatory and bone remodeling markers in gingival crevicular fluid following periodontal therapy concluded that except for an upregulation of IL-8, there seems to be a local immunosuppressant effect of smoking [3]. However, the role of those cytokines in smoking patients and how they mediate the host immune response as well as their effect on periodontal therapy, remains obscure.

In the process of diagnosing periodontal health, the outcomes of periodontal therapy and long-term prognosis are determined using clinical and radiographic surrogates. The shortcomings of clinical proxies are obvious as they cannot effectively detail the status of the disease, predict exacerbation and progression, or response to therapy. This is especially true for cigarette smokers with periodontitis [19]. Identification of biomarker profiles pre- and post-therapy may become a significant supplement to the clinical estimates in predicting disease progression. Therefore, expanding the knowledge around biomarkers presence and functionality in smokers and non-smokers with periodontitis may lead to a more profound understanding of the impaired response to periodontal therapy in smokers [20, 21].

To our knowledge a dearth of studies has presented and compared several serum cytokine levels in heavy smokers and non-smokers following periodontal therapy with an objective validation of smoking status in severe periodontitis patients. We hypothesize that at baseline (T0) higher levels of serum cytokines are observed in smokers with periodontitis compared with non-smokers with periodontitis and with decreasing intergroup differences along therapy (at T1 and T2). As levels of IL-8 and IL-6 appears to be impacted by heavy smoking following periodontal therapy [3, 22] the aims of this prospective cohort study were to determine the influence of smoking on the presence and level of serum inflammatory markers and clinical measures in smokers with stage III and IV periodontitis, and to compare levels of serum cytokines in smokers and non-smokers following step I-III and 12-month step IV periodontal therapy, with particular emphasis on IL-6 and IL-8.

## Methods

### Study design

This prospective longitudinal cohort study is based on a previous study conducted April 2012 through March 2015 [23]. The cohort was smokers (*n* = 40) and non-smokers (*n* = 40) with periodontitis, followed over 12 months after step I-III periodontal therapy. The study protocol was approved by the Institutional Medical Research Ethics Committee with registration 2011/151-6 (14/03/2011), University of Bergen, Norway and was conducted in accordance with the Helsinki Declaration of 1975, version 2013. Participating subjects read and signed the informed consent prior to inclusion in the study. The present follow-up study was approved by the Institutional Medical Research Ethics Committee with registration 120,868 (24/06/2020) and retrospectively registered in ClinicalTrials.gov with registration NCT05120206 (02/11/2021). All methods were carried out in accordance with STROBE guidelines and regulations.

### Study sample

#### Sample size

The outcome measures in the present study were defined as the difference in levels of biomarkers in smokers and non-smokers following periodontal therapy, with a particular emphasis on IL-6 and IL-8. The biomarker IL-8 was of special interest as it is upregulated in smokers following therapy, and the upregulation is present both in serum and in GCF [3, 24]. A significant correlation between cytokines in serum and GCF supports the hypothesis that the inflammatory reaction due to periodontitis is not restricted to diseased sites [25]. As GCF levels of IL-8 were present before and after periodontal therapy for this study sample, the sample size calculation was performed entering mean (pg/mL) in GCF and standard deviation for the primary cytokine IL-8. The calculation indicated that 30 participants per group would be sufficient to achieve a power of 80%, at a level of significance of 5%.

#### Smoking status

Subjects were classified as smokers (> 10 cigarettes/day for at least 5 years) and non-smokers (never smoked or not smoked the last 5 years). Heavy smoking status was objectively validated by measuring cotinine levels in serum at baseline [26]. The level of cotinine was determined using an enzyme linked immunosorbent assay (Cotinine ELISA Kit, MyBioSource, San Diego, CA, USA).

#### Study group

**Fig. 1:**
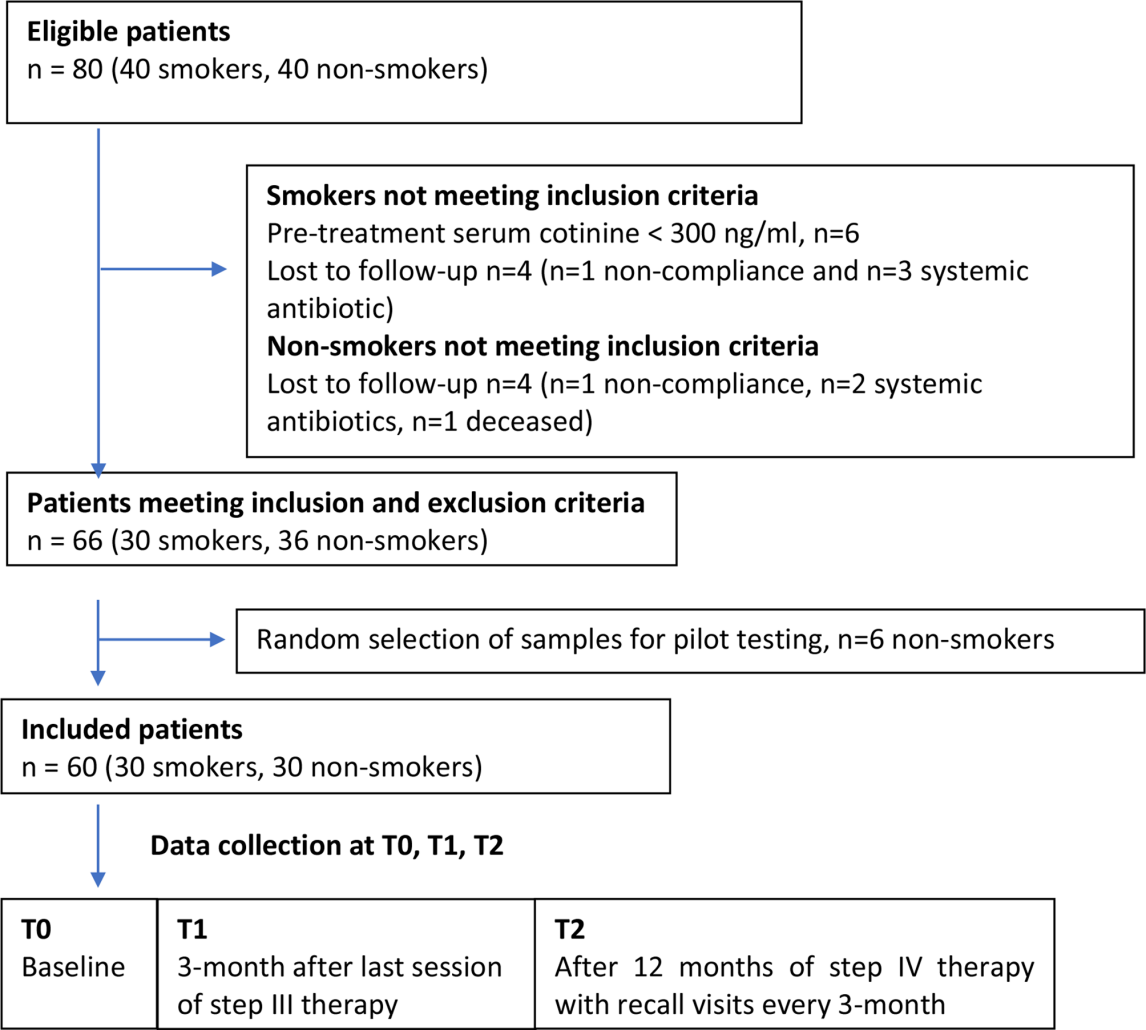
Flowchart of study patients and data collection

From the 80 eligible participants included in the original cohort study [23], 30 smokers and 30 non-smokers were enrolled in the present study (Fig. 1). The inclusion criteria were: (1) Healthy subjects between 35 and 75 years not using medication that could affect periodontal healing, (2) At least four non-adjacent teeth with interproximal probing depth (PD) ≥ 6 mm, clinical attachment loss (CAL) > 5 mm, and bleeding on probing (BoP) [27, 28], (3) Smokers with pre-treatment (T0) serum cotinine level ≥ 300 ng/mL [29] and non-smokers with T0 serum cotinine level < 15 ng/mL, (4) Subjects who completed data-collection at T0, T1, and T2.

The exclusion criteria were: (1) Subjects who had received subgingival instrumentation or systemic antibiotics within the last 6 months, (2) Presence of any current medical condition or use of medications affecting periodontal healing (including diabetes, cardiovascular diseases, and autoimmune diseases), (3) Use of snuff or incorrect reporting of smoking status, (4) A delay of scheduled visits by more than one month during phase IV therapy.

### Periodontal therapy

All included patients received periodontal therapy performed by one calibrated operator (DFB) [23, 30]. The overall therapy comprised a complete periodontal examination, oral hygiene motivation and instruction, non-surgical therapy, extraction of teeth with hopeless prognosis [31], and re-instrumentation and periodontal surgery (step I-III periodontal therapy) [32]. Surgical treatment was performed in patients with adequate oral hygiene and PD > 5 mm with BoP [33, 34] and comprised gingivectomy, access flap surgery, or regenerative therapy. The supportive periodontal care (SPC) lasted for 12 months and was executed at 3-month intervals (step IV periodontal therapy) in 60-minute appointments including re-motivation and re-instruction in oral hygiene, instrumentation, and full mouth plaque removal. Smokers were motivated for smoking cessation and encouraged to participate in a public smoking cessation program.

### Data collection

Clinical data and blood samples (serum) were collected by one calibrated examiner (DFB) at baseline pre-treatment (T0), 3 months after the last session of step I- III (T1), and after 12 months with step IV periodontal therapy (T2) [30] (Fig. 1). Data collection took place April 2012 through March 2015. Detailed medical, dental, periodontal, and smoking history was obtained from each patient through clinical examinations (including weight and height), health forms, questionnaires, and by consulting physicians.

For the purpose of this study, the following clinical parameters were reported: PD as the distance from the gingival margin to the probable base of the pocket; CAL as the distance from the cemento-enamel junction or the margin of a dental restoration to the probable base of the pocket; full mouth bleeding index (BI) as the percentage of sites showing bleeding on gentle probing (BoP) [35]; full mouth dental plaque index (PI) as the percentage of tooth surfaces with visible plaque following staining with disclosing solution [36]. PD and CAL were measured using a periodontal probe (PCPUNC 15, Hu-Friedy, Chicago, IL, USA) at six sites per tooth rounding up to the nearest mm. As a supplement to staining, the periodontal probe was used to discriminate between plaque and pellicle.

Peripheral blood samples were collected from each patient using venepuncture in the antecubital fossa following overnight fast. Vacutainer tubes containing anticoagulant were filled with blood at T0, T1, and T2. The tubes were preserved at bench site less than 2 h. Part of the blood sample was centrifuged for 10 min at 3000 g to produce 100 µL serum aliquots for each sample. The serum samples were stored at − 80 °C until analysis.

### Sample processing

The experimental laboratory procedures were at all times conducted by one pair of operators (YX and LK). The primary and specialized trained operator (YX) provided comprehensive training to LK. Based on inflammatory molecules involved in periodontal pathogenesis, blood serum levels of the following systemic cytokines were measured: IL-6, IL-8, High-sensitivity C-Reactive Protein (hs-CRP), IL-10, TNF-α, and IP-10. Measurements of IL-6, IL-8, IL-10, TNF-α, and IP-10 were determined using a custom Bio-Plex Human Pro^™^ Chemokine 5plx EXP assay kit (catalogue number LX10009222405, Bio-Rad, Hercules, CA, USA). Following manufacturer’s instructions, serum samples were diluted 1:1 with a sample dilution buffer and incubated with magnetic beads. After series of washing using an automated magnetic wash station (Bio-Plex Pro II, Bio-Rad), biotinylated conjugated antibody was added and then incubated with Streptavidin-Phycoerythrin conjugate as a fluorescent indicator. All bead readings were conducted using a Bio-plex 200 system® and fluorescence values were collected. The reader settings were RP1 low and DD Gates of 5000 (low) and 25 000 (high). The data acquisition was set to 50 beads per region. Prior to each measurement, the Bio-Plex 200 system® underwent calibration using a designated calibration kit (Bio-Rad, Hercules, CA, USA) to ensure accuracy and reliability of the readings. The calibration curve for each cytokine was analysed with five parametric logistic curve regressions using Bio-Plex manager software (ver 6.0, BioRad). The standard curve for each marker presents an overall range of 107,489 − 0.064 pg/mL. The quality control was run in the assay plate, and outliers were identified as standard data points that did not meet accuracy or precision requirements when performing curve fitting as a recovery range was 70–130%. The biomarker hs-CRP was measured by immunometric assay. The serum sample from one patient in the non-smoking group was discarded due to insufficient quality. The maximum desired false discovery rate was set to 1%.

### Statistical analysis

The primary outcome variables were defined as the differences in blood serum levels (pg/µL) of IL-6 and IL-8 between smokers and non-smokers following periodontal therapy. The secondary outcomes were the differences in levels of blood serum hs-CRP, IL-1β, IL-10, INF-γ, TNF-α, and IP-10 and differences in clinical measures (PD and CAL) over the same timeframe. For each marker, outliers were identified using the ROUT method in GraphPad Prism version 9.3.1 [37]. In brief, the dataset of each marker was imported into Graphpad Prism and the ROUT method was used with a Q cutoff of 1%. The output showed that it passed the D’Agostino-Pearson normality test, likely due to more sensitive comparison of datapoints to a Gaussian distribution, as well as correct filtering with ROUT.

For the descriptive statistics, continuous variables were presented as means with standard deviations (SD), while categorical variables were presented as frequencies with percentages. For the continuous clinical measures (PD and CAL) at site level, linear regression models with cluster robust variance estimates to correct for clustering within individuals and repeated measures over time were used. For the categorical measures at site level (BOP, plaque, and PD ≤ 4 mm with no BOP), logistic regression models with cluster robust variance estimates were applied. Shapiro–Wilk test was applied to test for normal distribution of continuous outcomes. The biomarkers measured at three different time points were analyzed using linear regression models with cluster robust variance estimates. To indicate the model fit, r-square (R^2^) for the linear models and pseudo r-square for the logistic regression analyses are presented. For categorical variables with more than two categories, p-values between categories were based on Scheffé’s adjustment for multiple comparisons. To account for possible confounding factors, adjusted regression models including significant variables at baseline were performed by two different models; Model 1 adjusted for mean PD and CAL at TO and Model 2adjusted for marital status, education, and body mass index (BMI). For each timepoint, estimated marginal means with standard errors (SEM) were presented for the continuous variables and marginal values for percentages with SEMs for the categorical values. Changes over time and differences between categories were shown as mean differences with SEMs. Changes over time and differences between categories were presented using odds ratios (OR) with 95% CIs.

For the heatmap, each cytokine was harmonized with non-smokers at T0 as standard reference (set as 1) and the relative differences colour-coded.

*P*-values less than *p* < 0.05 were considered statistically significant. All statistical analyses were performed using STATA version 16.1 (Stata Statistical Software: Release 16; Stata-Corp LP, ColleStation, TX, USA). Graphs were produced using STATA.

## Results

A total of 180 serum samples from 30 heavy smokers and 30 non-smokers were analysed at three timepoints (T0, T1, and T2) (Fig. 1).

For smokers at T0, mean packyear of cigarettes was 36 (SD = 18), and mean cotinine level was 498 mg/mL (SD = 172 mg/mL). Gender and age were homogeneously distributed between the groups (Table 1). Non-smokers showed a significantly higher mean BMI than smokers (*p* = 0.024). More non-smokers than smokers were cohabitants (*p* = 0.019) and had education > 9 years (*p* = 0.009). Ten smokers (33%) accepted to participate in the smoking cessation program, but none managed to quit smoking over the course of the study. In total 54 patients received periodontal surgery, 29 smokers and 25 non-smokers.

**Table 1:** Baseline patient related characteristics presented as mean (± SD) or number (percentage) stratified by smoking status

	Smokers (*n* = 30)	Non-smokers (*n* = 30)	Overall (*n* = 60)	*p*-level
**Mean Age (SD)** ^a^	58.7 (9.1)	57.5 (10.0)	58.1 (9.5)	0.628
**Sex (%)** ^b^				0.190
Male	10 (30%)	15 (50%)	25 (42%)	
Female	20 (66%)	15 (50%)	35 (58%)	
**Marital status (%)** ^b^				0.019
Married or cohabitant	18 (60%)	26 (87%)	44 (73%)	
Single	12 (40%)	4 (13%)	16 (27%)	
**Education (%)** ^b^				0.009
≤9 years^C^	22 (73%)	12 (40%)	34 (57%)	
>9 years^C^	8 (27%)	18 (60%)	26 (43%)	
**Mean BMI (SD)**Mean BMI (SD)^a^	24.0 (3.8)	25.8 (2.8)	24.8 (3.4)	0.024

^a^Two-sample independent t-test

^b^Chi-square test

^C^The 9 years cutoff was used because 9-year education is mandatory in Norway

At T0, T1, and T2 smokers had significantly higher mean PD (all *p* ≤ 0.001) and mean CAL (*p* = 0.006, *p* ≤ 0.001, and *p* = 0.002, respectively) compared with non-smokers (Table 2). In linear regression models significant differences were detected for mean PD and CAL over time in smokers and non-smokers; PD from T0 to T1 (both *p* < 0.001), T1 to T2 (*p* = 0.026 and *p* = 0.019), and T0 to T2 (both *p* < 0.001); CAL from T0 to T1 (both *p* < 0.001) and T0 to T2 (both *p* < 0.001) (Table 2). For both groups, logistic regression models revealed significant intragroup differences over time for BI and PI (both *p* < 0.001). The number of sites with PD≥4 mm without BOP differed significantly between smokers and non-smokers at T1 (*p* = 0.011) and over time; from T0 to T1 (both *p* < 0.001), T1 to T2 (*p* = 0.001 and *p* = 0.052), and T0-T2 (both *p* < 0.001) (Table 2).

**Table 2:** Patient-related clinical measures for 30 smokers compared with 30 non-smokers at T0, T1 and T2

	T0	P	T1	P	T2	*p*	Δ_T0−T1_	*p*	Δ_T1−T2_	*p*	Δ_T0−T2_	*p*
PD							Mean diff ± SEM		Mean diff ± SEM		Mean diff ± SEM	
Non-smokers	3.34 ± 0.08		2.31 ± 0.06		2.43 ± 0.06		1.02 ± 0.06	< 0.001	-0.12 ± 0.05	0.026	0.91 ± 0.06	< 0.001
Smokers	3.71 ± 0.08		2.65 ± 0.69		2.81 ± 0.08		1.06 ± 0.07	< 0.001	-0.16 ± 0.67	0.019	0.90 ± 0.71	< 0.001
Mean diff ± SEM	0.38 ± 0.11	0.001	0.34 ± 0.09	< 0.001	0.39 ± 0.11	0.001	0.04 ± 0.93	0.995	-0.05 ± 0.82	0.910	-0.01 ± 0.09	0.833
CAL							Mean diff ± SEM		Mean diff ± SEM		Mean diff ± SEM	
Non-smokers	3.95 ± 0.09		3.10 ± 0.08		3.17 ± 0.08		0.84 ± 0.67	< 0.001	-0.07 ± 0.06	0.212	0.78 ± 0.75	< 0.001
Smokers	4.39 ± 0.13		3.63 ± 0.10		3.63 ± 0.11		0.76 ± 0.10	< 0.001	0.00 ± 0.50	0.990	0.76 ± 0.10	< 0.001
Mean diff ± SEM	0.44 ± 0.16	0.006	0.53 ± 0.13	< 0.001	0.46 ± 0.14	0.002	-0.09 ± 0.12	0.996	0.07 ± 0.08	0.775	-0.01 ± 0.12	0.626
BI							OR (95% CI)		OR (95% CI)		OR (95% CI)	
Non-smokers	71.1% ± 2.6%		24.1% ± 2.0%		35.7% ± 1.9%		0.13 (0.09;0.18)	< 0.001	1.75 (1.33;2.30)	< 0.001	0.23 (0.16;0.32)	< 0.001
Smokers	74.9% ± 2.9%		26.9% ± 1.8%		31.5% ± 1.7%		0.12 (0.08;0.19)	< 0.001	1.25 (0.99;1.58)	0.064	0.15 (0.11;0.23)	< 0.001
OR (95% CI)	1.21 (0.82;1.80)	0.342	1.15 (0.88;1.52	0.311	0.83 (0.66;1.04)	0.097	0.95 (0.54;1.66)	0.977	0.68 (0.41;1.13)	0.178	0.72 (0.50;1.02)	0.071
PI							OR (95% CI)		OR (95% CI)		OR (95% CI)	
Non-smokers	63.8% ± 3.3%		24.3% ± 2.4%		37.9% ± 3.3%		0.18 (0.11;0.30)	< 0.001	1.90 (1.25;2.91)	0.001	0.35 (0.25;0.48)	< 0.001
Smokers	57.6% ± 3.4%		23.6% ± 2.4%		35.7% ± 4.0%		0.23 (0.15;0.33)	< 0.001	1.79 (1.30;2.47)	< 0.001	0.41 (0.26;0.63)	< 0.001
OR (95% CI)	0.77 (0.52;1.15)	0.201	0.96 (0.67;1.38)	0.826	0.90 (0.58;1.40)	0.654	1.24 (0.66;2.35)	0.700	0.94 (0.56;1.60)	0.963	0.17 (0.68;2.03)	0.776
PD ≤ 4 mm no BOP							OR (95% CI)		OR (95% CI)		OR (95% CI)	
Non-smokers	24.8% ± 2.2%		67.3% ± 2.2%		52.9% ± 3.2%		6.22 (4.51;8.58)	< 0.001	0.54 (0.37;0.81)	0.001	3.40 (2.13;5;41)	< 0.001
Smokers	19.1% ± 2.4%		57.9% ± 3.0%		51.9% ± 3.4%		5.83 (3.90;8.74)	< 0.001	0.79 (0.62;1.00)	0.052	4.59 (2.96;7.13)	< 0.001
OR (95% CI)	0.71 (0.48;1.05)	0.084	0.67 (0.49;0.91)	0.011	0.96 (0.67;1.39)	0.835	0.94 (0.56;1.57)	0.954	1.44 (0.91;2.29)	0.154	1.35 (0.72;2.55)	0.511

Probing depth (PD); Clinical attachment loss (CAL); Bleeding index (BI); Plaque index (PI); Marginal mean values and percentages are presented with standard errors. PD and CAL are presented in mm and analysed using linear regression models. BI and PI are presented as percentages, and differences and changes are presented as Odds ratios (OR) with 95% confidence intervals (CI), based on logistic regression analysis

**Fig. 2:**
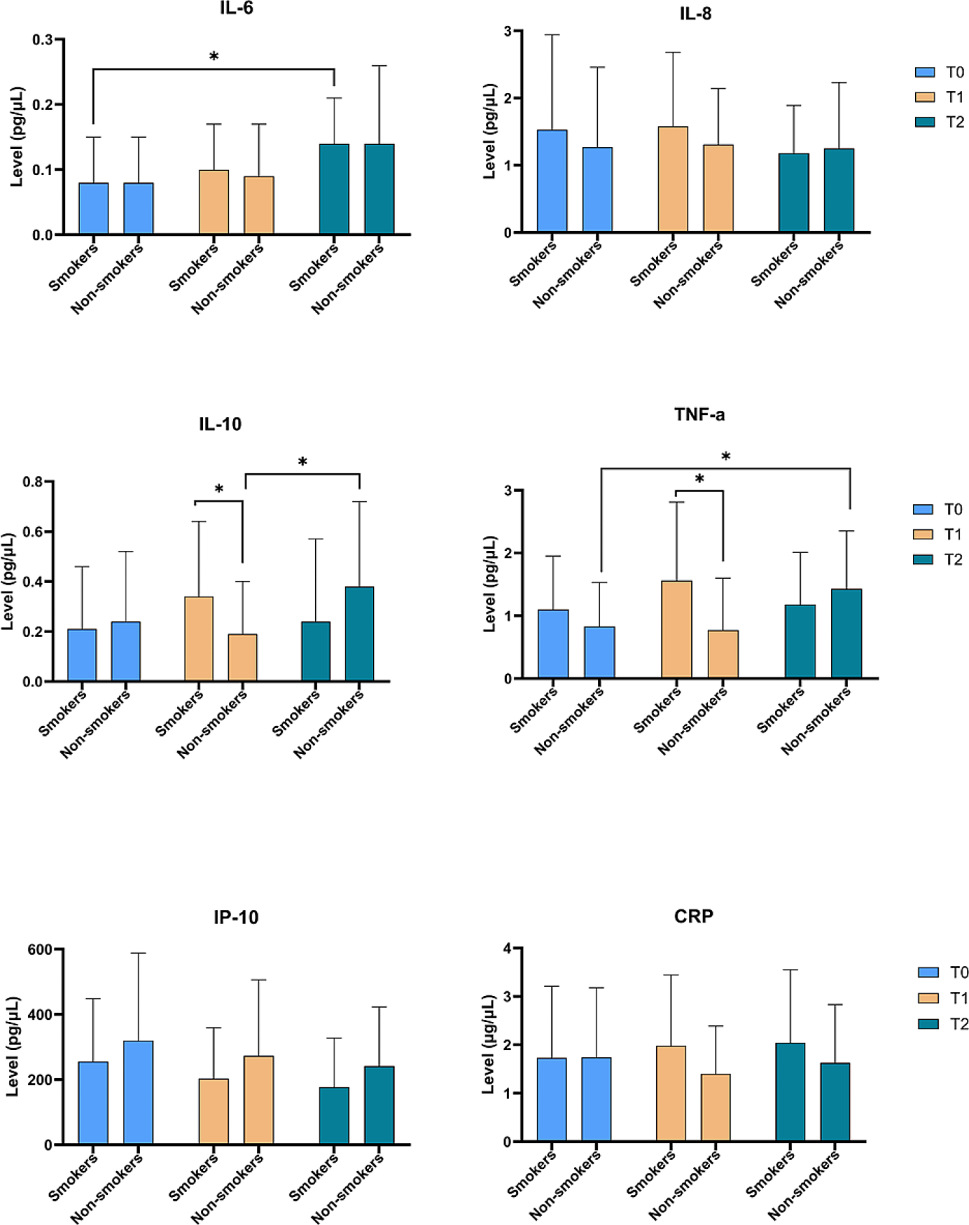
Bar graphs presenting the mean levels of serum biomarkers in smokers (*n* = 30) and non-smokers (*n* = 30) at T0, T1, and T2. *p < 0.05. See Table S1 for the mean level of each biomarker at T0, T1, and T2

The levels of serum cytokines are summarized in Fig. 2. The specific level of each biomarker in smokers and non-smokers at T0, T1, and T2 are presented in Table S1. At T1, smokers presented significantly higher levels of IL-10 (50.6%) and TNF-α (44.1%) compared with non-smokers (*p* = 0.037 and *p* = 0.007, respectively). Over time, IL-6 levels increased (75.0%) significantly in smokers from T0 to T2 (*p* = 0.004), TNF-α increased (73.0%) significantly from T0 to T2 in non-smokers (*p* = 0.026), and IL-10 increased (100.0%) significantly in non-smokers from T1 to T2 (*p* = 0.048). For IL-8 no significant changes were observed over time (all *p* > 0.05).

**Fig. 3:**
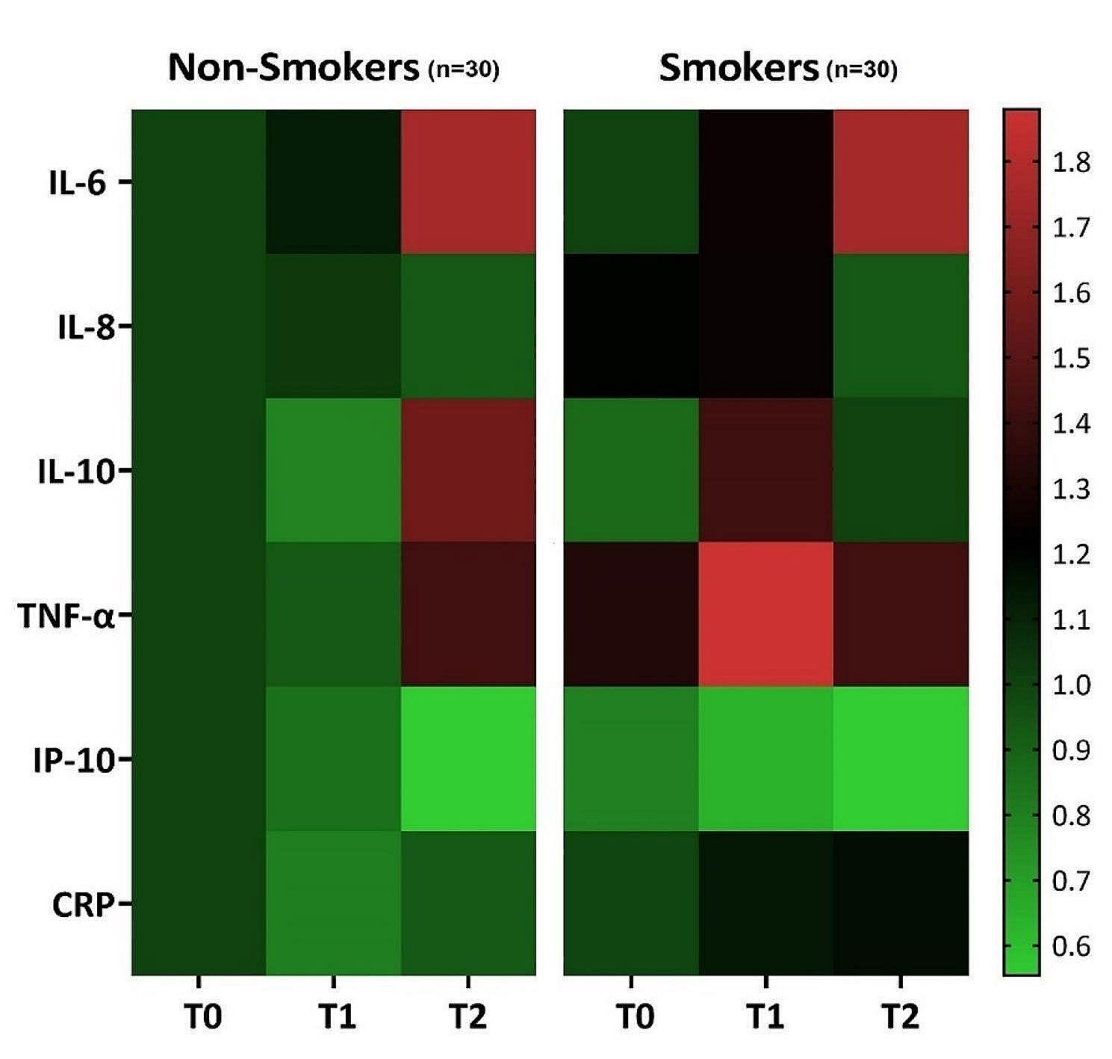
Heatmap of relative change in biomolecule concentrations (pg/µL for cytokines and µg/µL for CRP). At baseline (T0), after step I-III + 3 months healing (T1), and after 12 months with step IV therapy (T2)

From T1 to T2 the mean levels of IL-10 and TNF-α decreased in smokers and increased in non-smokers (Fig. 2), and the change over time difference in smokers compared with non-smokers is shown in Table 3. For IL-10 and TNF-α significant differences in change were detected in the unadjusted model (*p* = 0.030 and *p* = 0.017, respectively) and the adjusted models (a) (*p* = 0.036 and *p* = 0.018, respectively) and (b) (*p* = 0.021 and *p* = 0.032, respectively). The relative change in level of biomarkers is illustrated in Fig. 3.

**Table 3:** Differences in mean changes over time for levels of serum biomarkers in smokers (*n* = 30) compared with non-smokers (*n* = 30)

		Comparison	Mean difference* (95% CI)	*p*-value
**IL-6**				
	unadjusted	T1 – T0 =	0.008 (-0.042;0.059)	0.952
		T2 – T0 =	-0.003 (-0.064;0.059)	0.997
		T2 – T1 =	-0.011 (-0.078;0.056)	0.952
	adjusted^a^	T1 – T0 =	0.009 (-0.042;0.059)	0.947
		T2 – T0 =	-0.002 (-0.063;0.059)	0.998
		T2 – T1 =	-0.011 (-0.078;0.057)	0.953
	adjusted^b^	T1 – T0 =	0.009 (-0.043;0.061)	0.947
		T2 – T0 =	-0.008 (-0.070;0.054)	0.965
		T2 – T1 =	-0.017 (-0.085;0.051)	0.884
**IL-8**				
	unadjusted	T1 – T0 =	0.005 (-0.776;0.786)	0.999
		T2 – T0 =	-0.331 (-1.126;0.464)	0.718
		T2 – T1 =	-0.336 (-1.018;0.346)	0.630
	adjusted^a^	T1 – T0 =	0.011 (-0.775;0.797)	0.999
		T2 – T0 =	-0.320 (-1.117;0.477)	0.735
		T2 – T1 =	-0.331 (-1.015;0.353)	0.640
	adjusted^b^	T1 – T0 =	0.046 (-0.749;0.840)	0.994
		T2 – T0 =	-0.301 (-1.116;0.515)	0.771
		T2 – T1 =	-0.346 (-1.042;0.349)	0.624
**IL-10**				
	unadjusted	T1 – T0 =	0.180 (-0.009;0.369)	0.183
		T2 – T0 =	-0.108 (-0.310;0.095)	0.586
		T2 – T1 =	-0.288 (-0.494;-0.081)	**0.030**
	adjusted^a^	T1 – T0 =	0.180 (-0.010;0.370)	0.186
		T2 – T0 =	-0.106 (-0.315;0.104)	0.617
		T2 – T1 =	-0.286 (-0.497;-0.075)	**0.036**
	adjusted^b^	T1 – T0 =	0.182 (-0.008;0.372)	0.180
		T2 – T0 =	-0.126 (-0.333;0.082)	0.501
		T2 – T1 =	-0.308 (-0.517;-0.099)	**0.021**
**TNF-α**				
	unadjusted	T1 – T0 =	0.512 (-0.057;1.082)	0.220
		T2 – T0 =	-0.518 (-1.103;0.068)	0.232
		T2 – T1 =	-1.030 (-1.713;-0.347)	**0.017**
	adjusted^a^	T1 – T0 =	0.512 (-0.061;1.085)	0.224
		T2 – T0 =	-0.518 (-1.107;0.072)	0.236
		T2 – T1 =	-1.030 (-1.718;-0.342)	**0.018**
	adjusted^b^	T1 – T0 =	0.514 (-0.067;1.096)	0.232
		T2 – T0 =	-0.413 (-0.974;0.149)	0.361
		T2 – T1 =	-0.927 (-1.598;-0.256)	**0.032**
**IP-10**				
	unadjusted	T1 – T0 =	24.958 (-54.441;104.358)	0.828
		T2 – T0 =	30.324 (-58.002;118.651)	0.798
		T2 – T1 =	5.366 (-67.843;78.575)	0.990
	adjusted^a^	T1 – T0 =	26.282 (-55.058;107.621)	0.819
		T2 – T0 =	33.339 (-57.683;124.361)	0.774
		T2 – T1 =	7.057 (-66.007;80.121)	0.982
	adjusted^b^	T1 – T0 =	14.221 (-64.400;92.841)	0.939
		T2 – T0 =	20.729 (-66.204;107.663)	0.897
		T2 – T1 =	6.509 (-67.839;80.857)	0.985
**CRP**				
	unadjusted	T1 – T0 =	0.594 (-0.273;1.460)	0.412
		T2 – T0 =	0.425 (-0.546;1.396)	0.694
		T2 – T1 =	-0.169 (-0.891;0.553)	0.901
	adjusted^a^	T1 – T0 =	0.590 (-0.281;1.462)	0.420
		T2 – T0 =	0.426 (-0.551;1.402)	0.696
		T2 – T1 =	-0.165 (-0.893;0.564)	0.907
	adjusted^b^	T1 – T0 =	0.547 (-0.338;1.432)	0.484
		T2 – T0 =	0.364 (-0.629;1.358)	0.774
		T2 – T1 =	-0.183 (-0.917;0.551)	0.888

* Change for smokers compared with non-smokers

Interleukin 6 (IL-6), Interleukin 8 (IL-8) High-sensitivity C-reactive Protein (CRP), Interleukin 10 (IL-10), Tumor necrosis factor alfa (TNF-α), and Interferon gamma-induced protein 10 (IP-10).

Baseline (T0), after primary treatment + 3 months healing (T1), and after 12 months with supportive periodontal therapy (T2)

a: adjusted for mean PD and CAL at T0

b: adjusted for marital status, education, and BMI (body mass index)

## Discussion

The aims of this prospective cohort study were to determine the effect of smoking on the presence of inflammatory mediators in serum comparing serum cytokine levels in smokers and non-smokers with periodontitis stage III and IV following periodontal therapy. Differences in biomarker levels were observed between smokers and non-smokers indicating a modulating effect of smoking on systemic immunological responses in patients with severe periodontitis. The inflammatory profile in smokers was upregulated, and smokers and non-smokers showed a different systemic inflammatory response to periodontal therapy.

The therapeutic implication of a systemic inflammatory dysregulation in smokers with periodontitis are not fully understood, but the present study aligns with previous reports suggesting that smoking has a systemically induced effect on clinical outcomes following periodontal therapy [38–40]. At all observation points, significantly increased mean PD and mean CAL were found in smokers, demonstrating that the more advanced periodontal baseline situation persisted over the course of study. Our observations also concur with a recent systematic review and meta-analysis concluding that smoking had a negative effect on non-surgical periodontal therapy with a lesser reduction in PD and CAL [40]. However, as in the present study, both smokers and non-smokers responded to treatment with a significant reduction in PD and CAL.

Smoking appears to trigger an overall increased systemic inflammatory response [41]. The present study suggests that the inflammatory response in smokers is not linked to either proinflammatory or regulatory cytokines, but rather to an overall amplified systemic inflammatory response. The levels of cytokines in smokers seem less influenced by treatment induced changes in PD, CAL, BI, and PI compared with non-smokers. Increased serum levels of inflammatory markers are associated with early clinical signs of periodontal breakdown [42] and an altered inflammatory profile in smokers may predict disease progression [19]. Therefore, the significantly different serum cytokine levels between smokers and non-smokers shown in this study might be linked to impaired clinical outcomes following periodontal therapy in smokers.

Proinflammatory cytokines, predominantly produced by activated macrophages, take part in the upregulation of inflammatory reactions [43]. They play a critical role in the pathogeneses of periodontitis modulating the inflammatory response leading to soft tissue destruction and bone resorption [10]. IL-6 is considered a marker for periodontal disease activity [2, 44], most likely a consequence of systemic disruption of bacteria or bacterial products. In the present study IL-6 levels increased from T0 to T2 in non-smokers and smokers, reaching a peak during supportive periodontal therapy. Systemic IL-6 may also be expected to decrease following periodontal therapy [18]. IL-6 commands dual properties inducing bone resorption [45] and promoting soft tissue formation [44, 46]. A balancing function inhibiting IL-1β and TNF-α production has also been presented [2, 44, 45]. Hence, soft tissue formation over the course of therapy could in part explain an increased expression of IL-6 during periodontal healing. Indeed, a previous study investigating local inflammation in gingival crevicular fluid using the same cohort of patients observed increased IL-6 levels following step IV therapy in smokers and non-smokers [3].

Similar levels of IL-6 and IL-8 in smokers and non-smokers indicate that smoking has no major impact on systemic serum concentrations in severe periodontitis patients. However, it has been shown that smoking can augment the production of IL-6 and IL-8 [24, 39, 47]. Nicotine, a major component of tobacco smoke, may increase IL-8 production in gingival epithelial cells and impair IL-8 agonists in neutrophils [39, 48]. Thus, IL-8 might play a crucial role in the local periodontal inflammatory defense and be of significance for impaired treatment outcome and recurrence of periodontitis in smokers. The increase in IL-8 concentration in smokers might, however, be a local upregulation having no or limited systemic effect.

Patients with chronic diseases might present with elevated TNF-α plasma levels due to a modulated immune response [49]. The same could be true for smokers [41, 50], in particular smokers with periodontitis demonstrate elevated TNF-α plasma levels [51]. The present study observed elevated TNF-α levels in smokers at T1, but only non-smokers showed a significant reduction following periodontal therapy also reported in other recent work [52]. The difference in change of TNF-α levels between smokers and non-smokers following step IV therapy implicates TNF-α as a key inflammatory mediator for periodontitis in both smokers and non-smokers. However, in non-smokers changes in TNF-α levels over time mirror clinical responses to periodontal therapy, whereas this association was less expressed in smokers [53, 54].

IL-10 is an immunoregulatory cytokine critical to the balance between microbial and inflammatory responses in periodontitis [55]. IL-10 actively suppresses the secretion of proinflammatory mediators [56] and is an inhibitor of macrophage and T-cell effector function [57]. At T1, a significantly elevated level of IL-10 was observed in smokers, while during step IV therapy IL-10 increased only in non-smokers. In non-smokers, increased level of IL-10 may occur as a consequence of increased BI and the association is most likely related to progression and/or recurrence of periodontitis during step IV therapy. In contrast, ex-vivo cell cultures show that smokers with periodontitis express a lower IL-10 level than non-smokers [58]. The same appears true for IL-10 in gingival crevicular fluid [3, 59]. The consistent findings of reduced levels of IL-10 in non-smokers demonstrate that IL-10 may elicit a dysregulated immune response to periodontal disease progression in smokers.

IP-10 plays a key role in leukocyte homing to inflamed tissue and activation of Receptor Activator of Nuclear factor Kappa-Β ligand (RANKL) expression responsible for alveolar bone resorption [60]. A reduced expression of IP-10 in smokers compared with never smokers has been shown in human lung cells [61]. In periodontitis patients IP-10 has been detected in higher levels in serum, saliva, and gingival crevicular fluid compared with healthy patients [62], whereas aggressive forms of periodontitis have been associated with lower systemic levels [11]. An observed decrease in IP-10 levels in smokers over the course of periodontal therapy, might indicate that IP-10 contributed to perpetuation of inflammation and thus to tissue damage via RANKL in smokers.

CRP is generally associated with higher levels in smokers [41], and severe periodontitis is associated with increased CRP serum levels compared with otherwise healthy patients [63]. No significant difference in CRP levels was found between non-smokers and smokers in the present study. Generally, it is challenging to evaluate systemic CRP levels in non-smokers and smokers because both smoking and periodontitis may stimulate increased CPR levels. A high-sensitive CRP (hs-CRP) analysis can, however, more likely detect changes in the expression of general inflammation in response to periodontal therapy [64].

Smoking is a broadly accepted periodontitis risk factor [38, 65]. Recording serum cotinine concentrations [3], the present study objectively and accurately validated heavy smoking and non-smoking status in included patients [66, 67]. Still, smoking status is often subjectively reported, an approach prone to bias [67] as the reporting of quantity and type of smoking appears incomplete, a key limitation in many studies [68]. Other strengths of the present study are the inclusion of patients with severe periodontitis and the prospective study design. Establishing clear definitions of periodontitis and smoking status also dismisses the notion that smoking-associated relative risk may be dependent on disease definition [69].

As the present study is a prospective cohort study of healthy individuals of either heavy smokers or not smokers, the participants are to some extent two extremes and the distribution of confounders between the non-smoking and smoking groups are not at random. This selection bias should therefore be taken into consideration in the interpretation of the findings. Intergroup differences are adjusted for in the regression analyses. The statistical power in the adjusted regression analyses must be considered as moderate. In contrast, the longitudinal design, with data collected at three timepoints, increases the statistical power. Moreover, the individual role of a cytokine in a complex chronic disease including periodontitis must be tempered within the room of its complexity [44]. That a single cytokine may govern several proinflammatory and regulatory functions while influencing other cytokines [70], makes it challenging to interpret their regulatory networks. Also, it is challenging to adjust for patient and site related factors impacting levels of systemic cytokines. In the present study, higher BMI in non-smokers could have influenced the levels of biomarkers [71, 72]. Also, it is challenging to adjust for individual patient and site related factors and variations in therapeutic approaches. However, the high sensitivity and accuracy offered by the Bio-Plex technology broadens the understanding of the complex immune reaction in periodontitis and other diseases [73, 74]. Samples were collected at three timepoints over the course of the study, and despite efforts to standardize all procedures, deviations are to be expected. Some of the serum samples were stored up to 8 years prior to analysis, but still within the time range of reliable outcomes according to the manufacturer. Some degradation, however, of the sample material during the storing period might be expected. Additionally, because of the low-grade inflammation presented in periodontitis [75], the Bio-Plex technology seemed to reach its capacity to accurately measure systemic cytokines in patients with periodontal disease. As such, it may be necessary to apply other more sensitive or thorough screening methods to accurately determine systemic biomarkers in periodontal patients. In the future a complete genomics analysis combined with bioinformatics may promote discovery of new biomarkers as well as updated regulatory systems to help with the validation of biomarkers and their successful translation into clinical practice.

## Conclusion

Serum levels of IL-6 and IL-8 in periodontitis patients do not seem to be influenced by smoking. Higher serum levels of TNF-α and IL-10 in smokers following step III periodontal therapy indicate a systemic inflammatory dysregulation effect of smoking. Thus, the present study supports previous observations that smoking induces immunological responses that might have a negative impact on the outcome of periodontal therapy.

### Electronic supplementary material

Below is the link to the electronic supplementary material.

Supplementary Material 1

## Data Availability

The data that support the findings of this study are available from the corresponding author upon reasonable request.

## Abbreviations

IL: Interleukin
IP: Induced protein
TNF-α: tumor nekrosefaktor-α
Hs-CRP: High sensitive C-reactive protein
GCF: Gingival crevicular fluid
BMI: Body mass index
PD: Probing depth
BoP: Bleeding on probing
CAL: Clinical attachment level
SPC: Supportive periodontal care
