# Supplementary material for: Serum biomarker levels in smokers and non-smokers following periodontal therapy. A prospective cohort study
Source: BMC Oral Health. 2024 Apr 16;24:463. doi: 10.1186/s12903-024-04196-8 (PMC11020793; doi:10.1186/s12903-024-04196-8)
Supplement: Supplementary file 1 — Supplementary Material 1 [file 12903_2024_4196_MOESM1_ESM.docx]

| **Table S1.** Mean (±SEM) levels of serum biomarkers at T0, T1, and T2 in smokers (n=30) and non-smokers (n=30) | | | | | | | | | |
| --- | --- | --- | --- | --- | --- | --- | --- | --- | --- |
|  | **T0** | | | **T1** | | | **T2** | | |
|  | **Smokers** | **Non-smokers** | ***p*** | **Smokers** | **Non-smokers** | ***p*** | **Smokers** | **Non-smokers** | ***p*** |
| IL-6 (R^2^=63%) | 0.08 ± 0.07 | 0.08±0.07 | 0.842 | 0.10 ± 0.7 | 0.09 ± 0.08 | 0.559 | 0.14 ± 0.07 **^B^** | 0.14 ± 0.12 | 0.969 |
| IL-8 (R^2^=63%) | 1.53 ± 1.41 | 1.27 ± 1.19 | 0.470 | 1.58 ± 1.10 | 1.31 ± 0.83 | 0.323 | 1.18 ± 0.71 | 1.25 ± 0.98 | 0.751 |
| IL-10 (R^2^=48%) | 0.21 ± 0.25 | 0.24 ± 0.28 | 0.683 | 0.34 ± 0.30 | 0.19 ± 0.21 | **0.037** | 0.24 ± 0.33 | 0.38 ± 0.34 **^A^** | 0.131 |
| TNF-α (R^2^=63%) | 1.10 ± 0.85 | 0.83 ± 0.70 | 0.182 | 1.56 ± 1.25 | 0.77 ± 0.83 | **0.007** | 1.18 ± 0.83 | 1.43 ± 0.92 **^B^** | 0.301 |
| IP-10 (R^2^=61%) | 255.20 ± 192.80 | 319.98 ± 268.39 | 0.128 | 203.32 ± 155.69 | 273.14 ± 233.10 | 0.188 | 177.16 ± 150.06 | 241.61 ± 181.41 | 0.158 |
| CRP (R^2^=64%) | 1.73 ± 1.48 | 1.74 ± 1.44 | 0.976 | 1.98 ±1.46 | 1.40 ±0.99 | 0.089 | 2.04 ± 1.51 | 1.63 ± 1.20 | 0.260 |
| Interleukin 6 (IL-6), Interleukin 8 (IL-8) High-sensitivity C-reactive Protein (CRP), Interleukin 10 (IL-10), Tumor necrosis factor alfa (TNF-α), and Interferon gamma-induced protein 10 (IP-10).  Baseline (T0), after primary treatment + 3 months healing (T1), and after 12 months with supportive periodontal therapy (T2).  Values for IL-6, IL-8, IL-10, TNF-α, and IP-10 are given in pg/μL, whereas values for CRP are shown in μg/μL. R^2^ is the explained variance from the models.  Letters in subscript to the mean denotes a significant (p<0.05) increase over time in smokers and non-smokers. A: T1 to T2 and B: T0 to T2 | | | | | | | | | |
